# Supplementary material for: YmoA functions as a molecular stress sensor in Yersinia
Source: Commun Biol. 2025 Feb 13;8:225. doi: 10.1038/s42003-025-07675-y (PMC11825884; doi:10.1038/s42003-025-07675-y)
Supplement: Supplementary file 2 — Supplementary Information [file 42003_2025_7675_MOESM2_ESM.pdf]

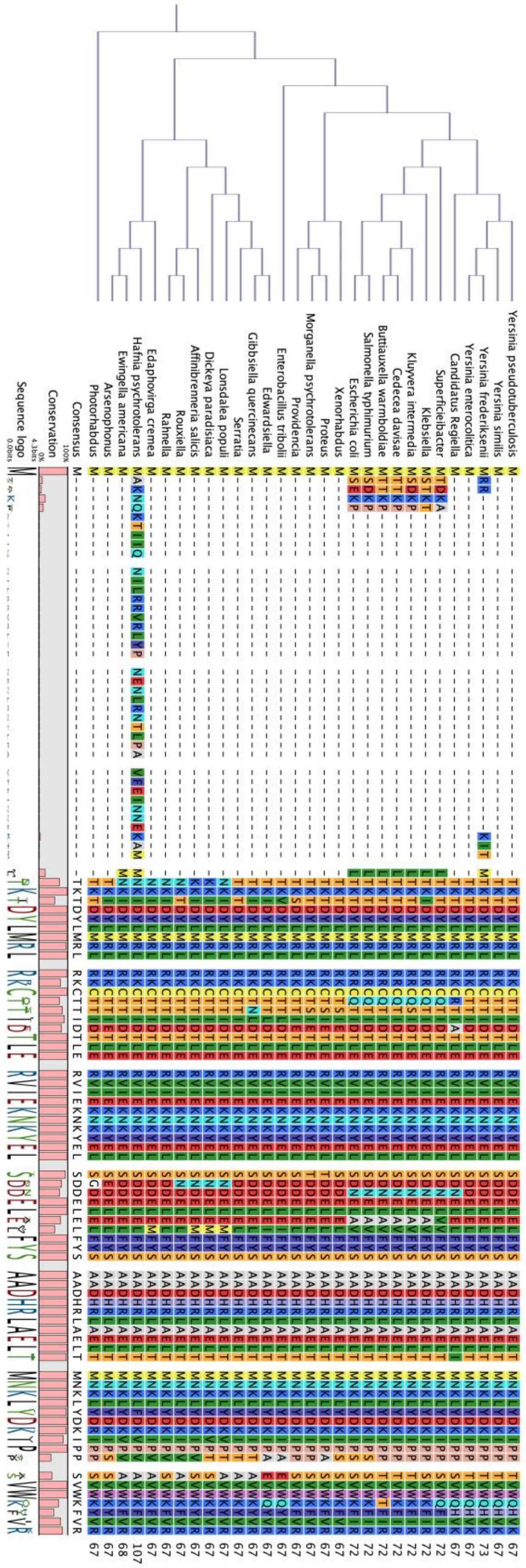

Figure S1. YmoA/Hha is a well conserved family of protein. Amino acids sequence alignment of YmoA/Hha gene from 30 species.

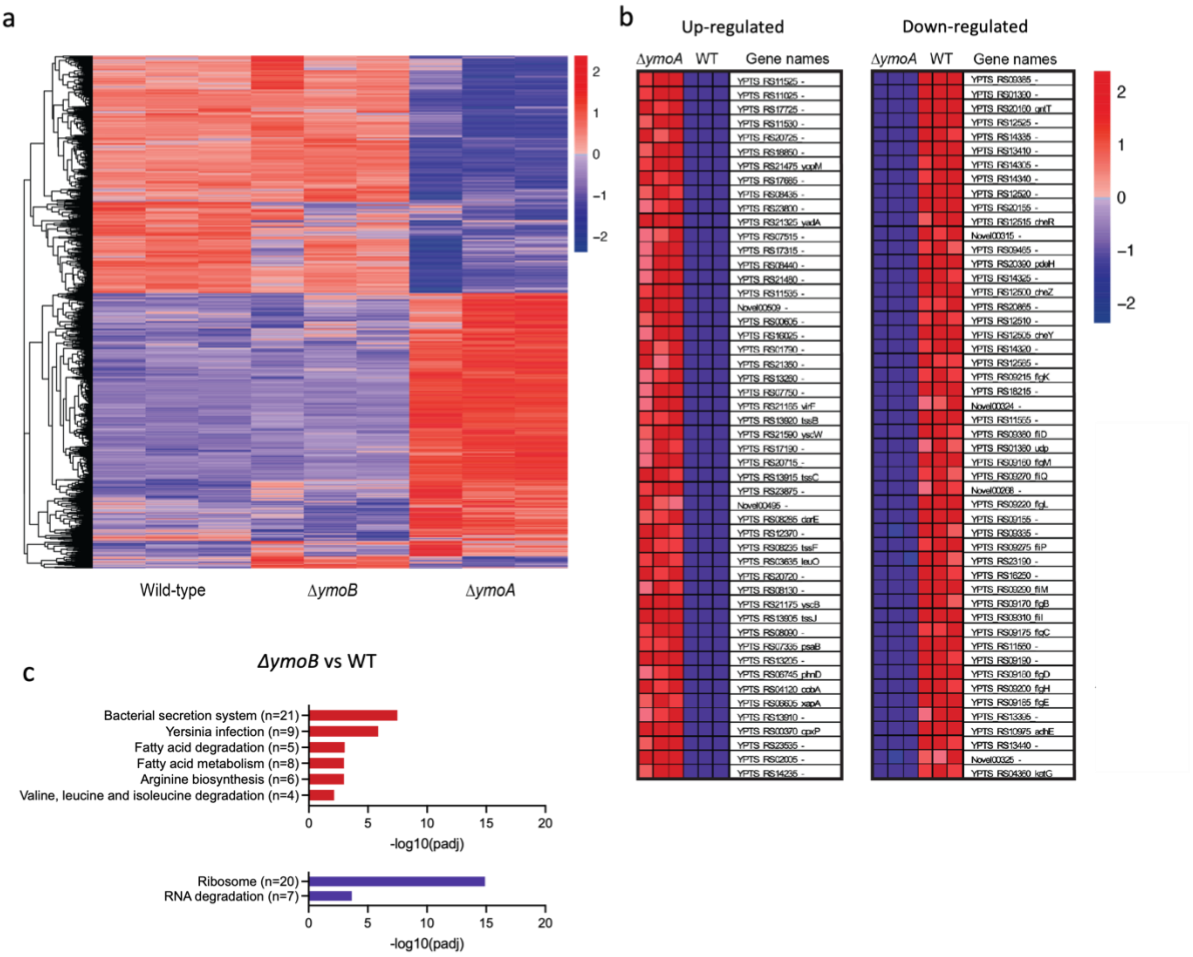

Figure S2. RNA-seq analysis demonstrates up-regulation of T3SS genes and down-regulation of flagellar genes in deletion mutants *ymoA* and *ymoB*. a-b. Clustering heat map of genes**.** Red shows genes with high expression levels, purple shows genes with low expression levels. The red to blue color range represents the $\log_2$ (FPKM+1) value from large to small. **a.** Overall results of FPKM cluster analysis, clustered using the $\log_2$ (FPKM+1) value. **b.** Genes with high phenotype correlation, genes in descending order by the correlation of phenotypes according to signal2noise. Left panel, 20 most up-regulated genes. Right panel, 20 most down-regulated genes. **c.** Significantly enriched terms in the GO enrichment analysis. Upregulated genes are shown on the top in red and downregulated genes are shown on bottom in purple.

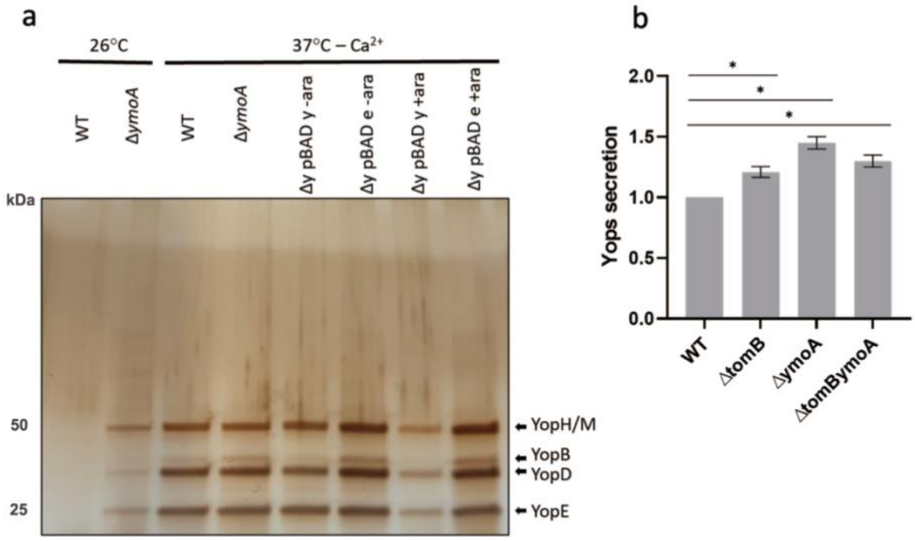

Figure S3: Yop secretion. a. Yops secretion of trans-complemented Δ*ymoA*. The same samples of bacterial supernatants as in Figure 2f were run on a SDS-page and silver-stained subsequently (n=1). b. Yop secretion of Figure 2f was quantified at T3SS inductive conditions (37°C -Ca2+) as the sum of YopE, YopD and YopH/M bands for each sample, normalized against the wild-type. The data shows the mean and SEM (n=3). Statistical analysis was done with a One-sample t-test (* $p \leq 0.05$).

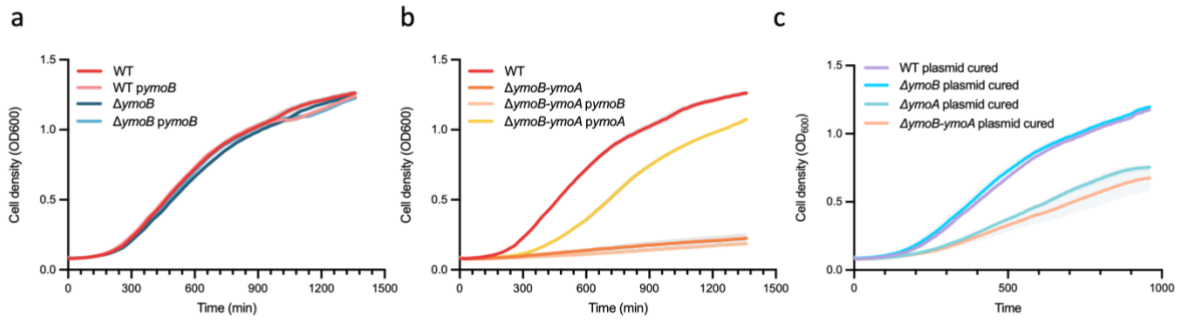

Figure S4. Most of *ymoA* deletion fitness cost comes from the plasmid and *ymoB* has no effect on bacterial fitness. a-c. Strains were grown at 26°C for 5h. Growth were determined by measuring the OD_{600}. The data represent the mean ± SD (n=3).

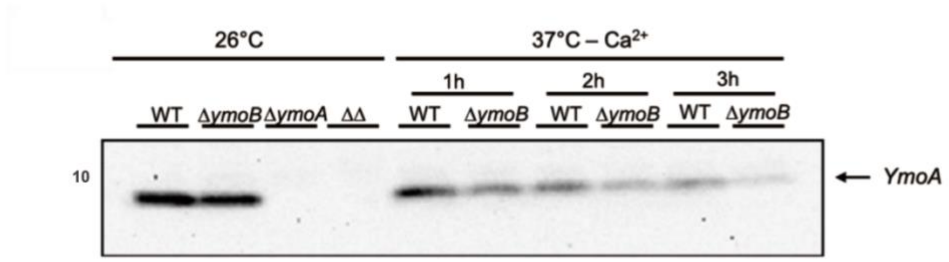

Figure S5. YmoB reduces YmoA degradation at 37°C. Western blots were performed on centrifuged pellet (whole cell) probed with anti-YmoA antibodies. Strains were grown at 26°C for 2h, subsequentially shifted to T3SS inductive conditions: (37°C - Ca^{2+}), and incubated for an additional 1-3 h. Shown is one representative Western Blot from one out of 3 biological replicates.

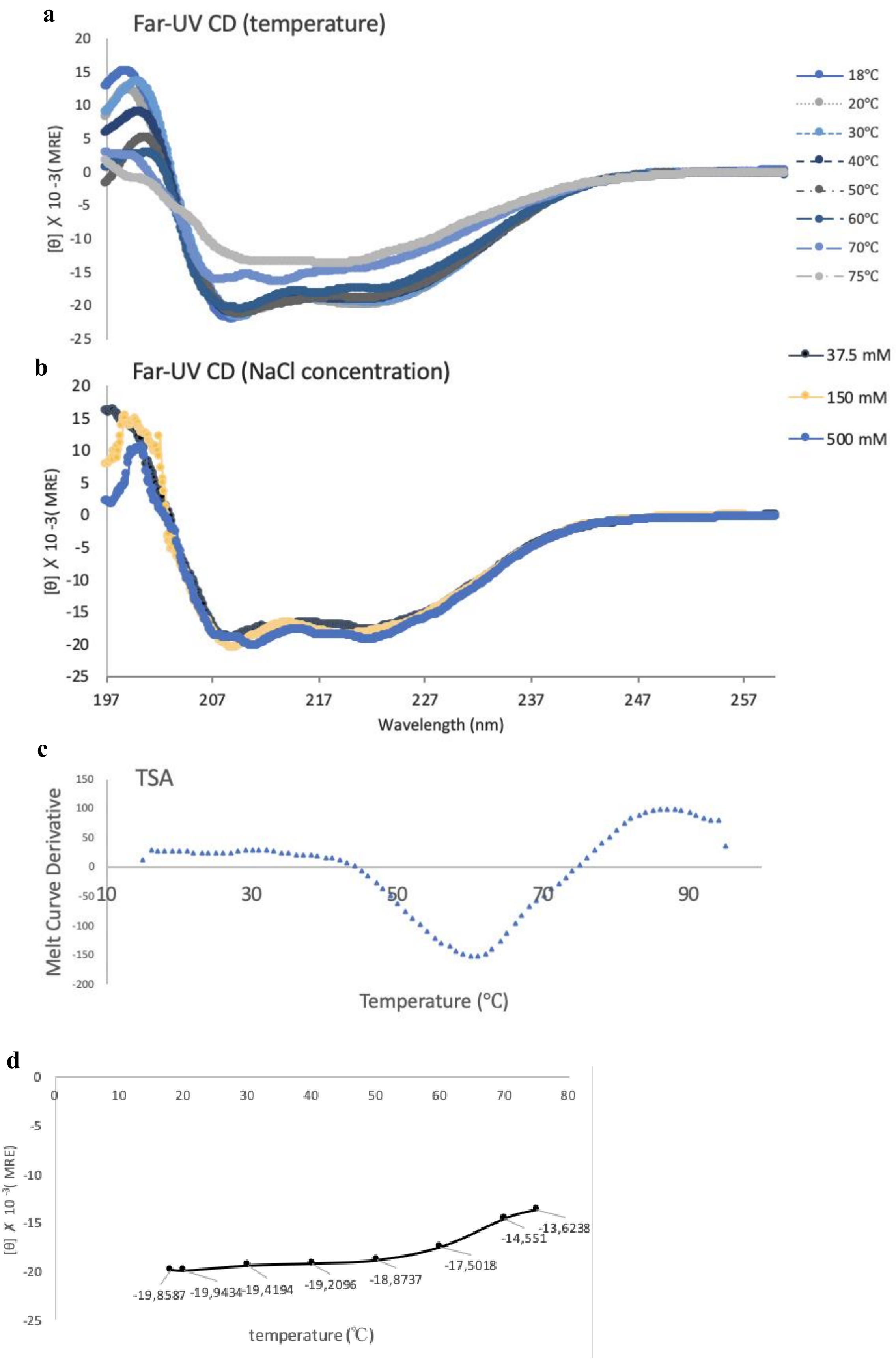
a
Far-UV CD (temperature)
[θ] X 10 -3(MRE)
18°C
20°C
30°C
40°C
50°C
60°C
70°C
75°C
b
Far-UV CD (NaCl concentration)
37.5 mM
150 mM
500 mM
Wavelength (nm)
c
TSA
Melt Curve Derivative
Temperature (°C)
d
-19,8587
-19,9434
-19,4194
-19,2096
-18,8737
-17,5018
-14,551
-13,6238
temperature (℃)

Figure S6. YmoA is well folded and stable. Both far-UV CD and thermal shift assay experiments confirmed that YmoA is folded, and stable at biological temperatures. **a.** CD spectra show YmoA continuously loses the second structure over the increase of temperature from 18 to 75 °C. **b.** YmoA possessed the same folding throughout the NaCl titration from 37.5 mM to 500 mM NaCl. **c.** Thermal shift assay also shows YmoA is folded below 50 °C and starts to unfold drastically at 60 °C. **d**. Far-UV CD signal of YmoA at 222 nm at 37.5 mM NaCl concentration was plotted as a function of temperature.

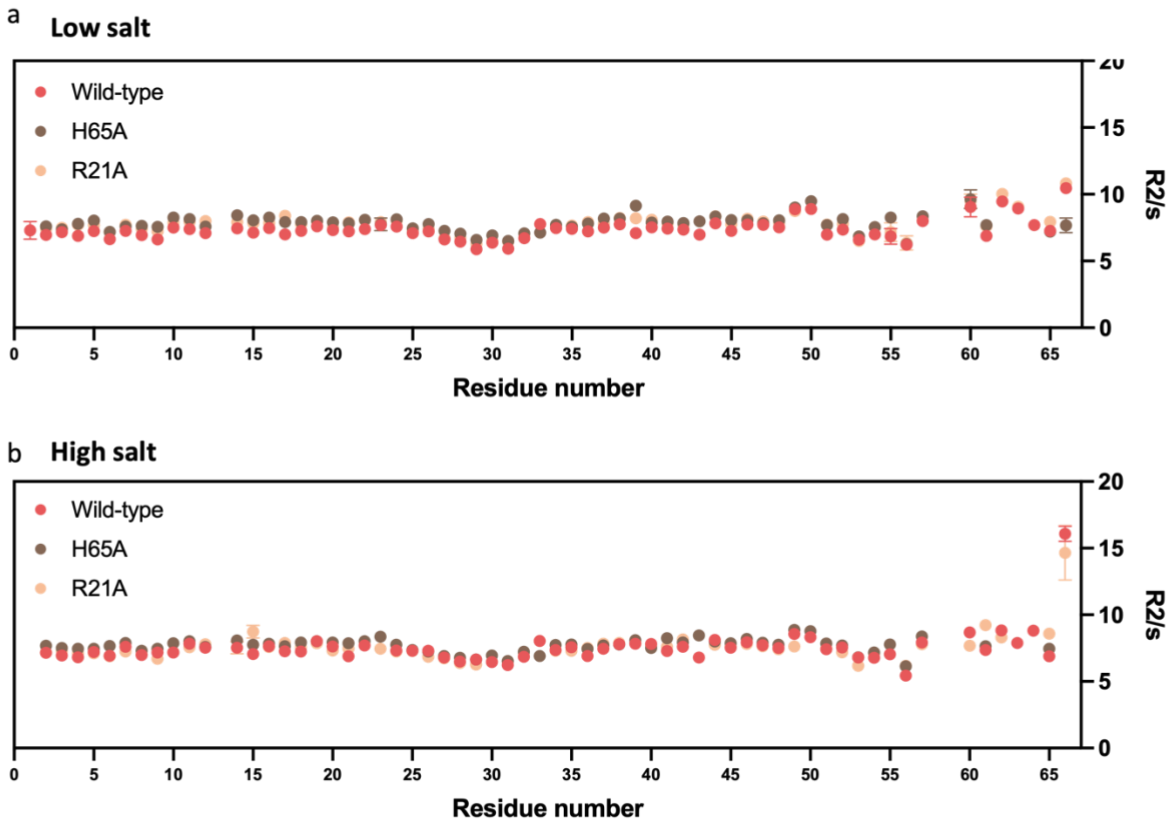

Figure S7. High salt broadens YmoA Lys-67 relaxation time. a-b. R2 relaxation measurement of the different YmoA residues in **a.** low salt 75mM or **b.** high salt 500mM concentration.

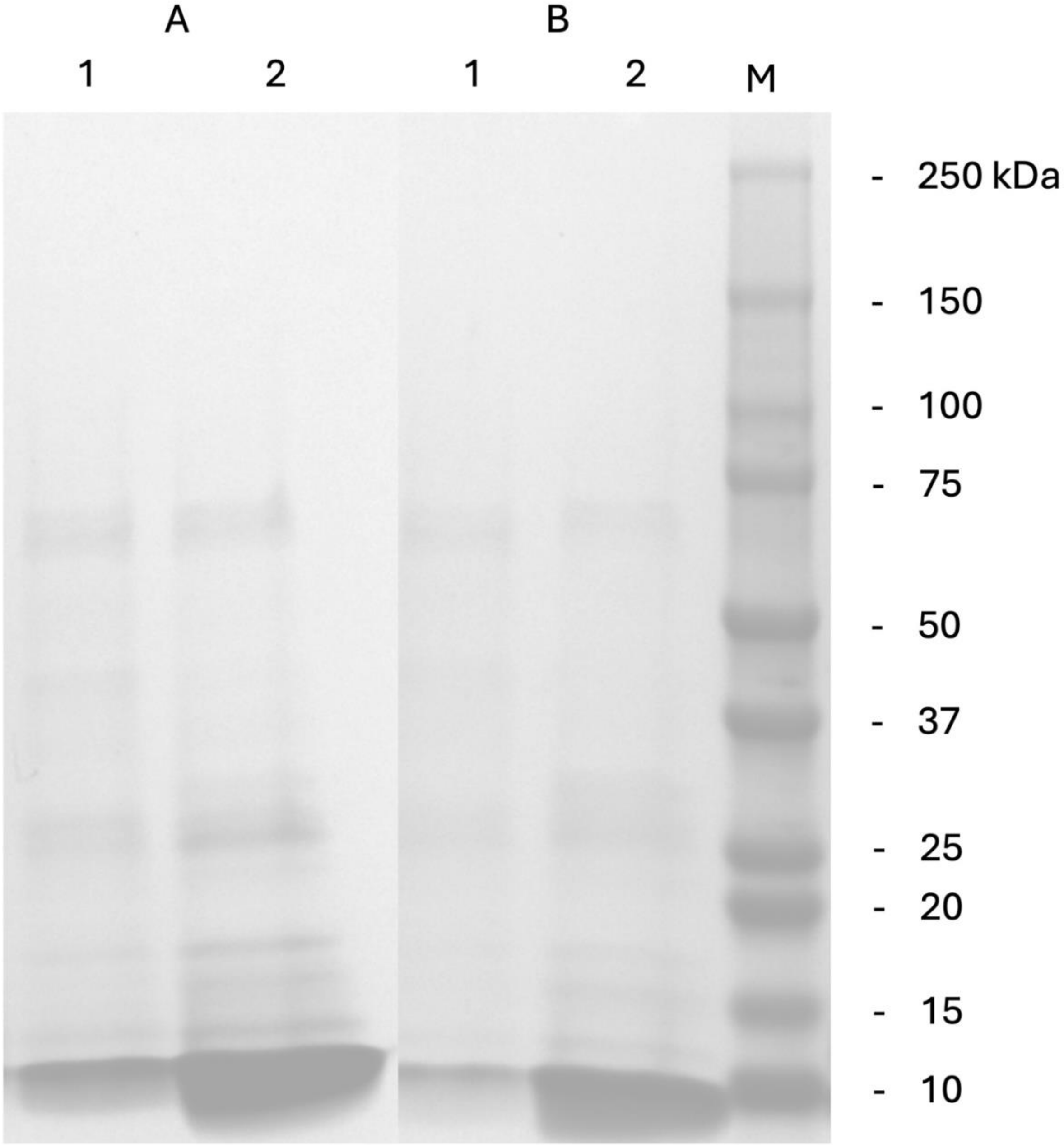

Figure S8. Two mutants, YmoA_R21A, YmoA_H65A (9 kDA), were purified in IMAC and were stable after concentrating. Samples for YmoA_R21A (A) and YmoA_H65A (B) were analyzed by SDS-PAGE. Lane 1, the elution fraction from IMAC purification; Lane 2, concentrated elution fraction. The elution fraction was concentrated to roughly 2.5 mg/ml, and stored at 4°C overnight; Lane M, molecular weight marker (Precision Plus Protein Dual Color Standards, Bio-Rad)

Fig S9. The uncropped Western blots of Fig 2e upper panel whole cell fraction.

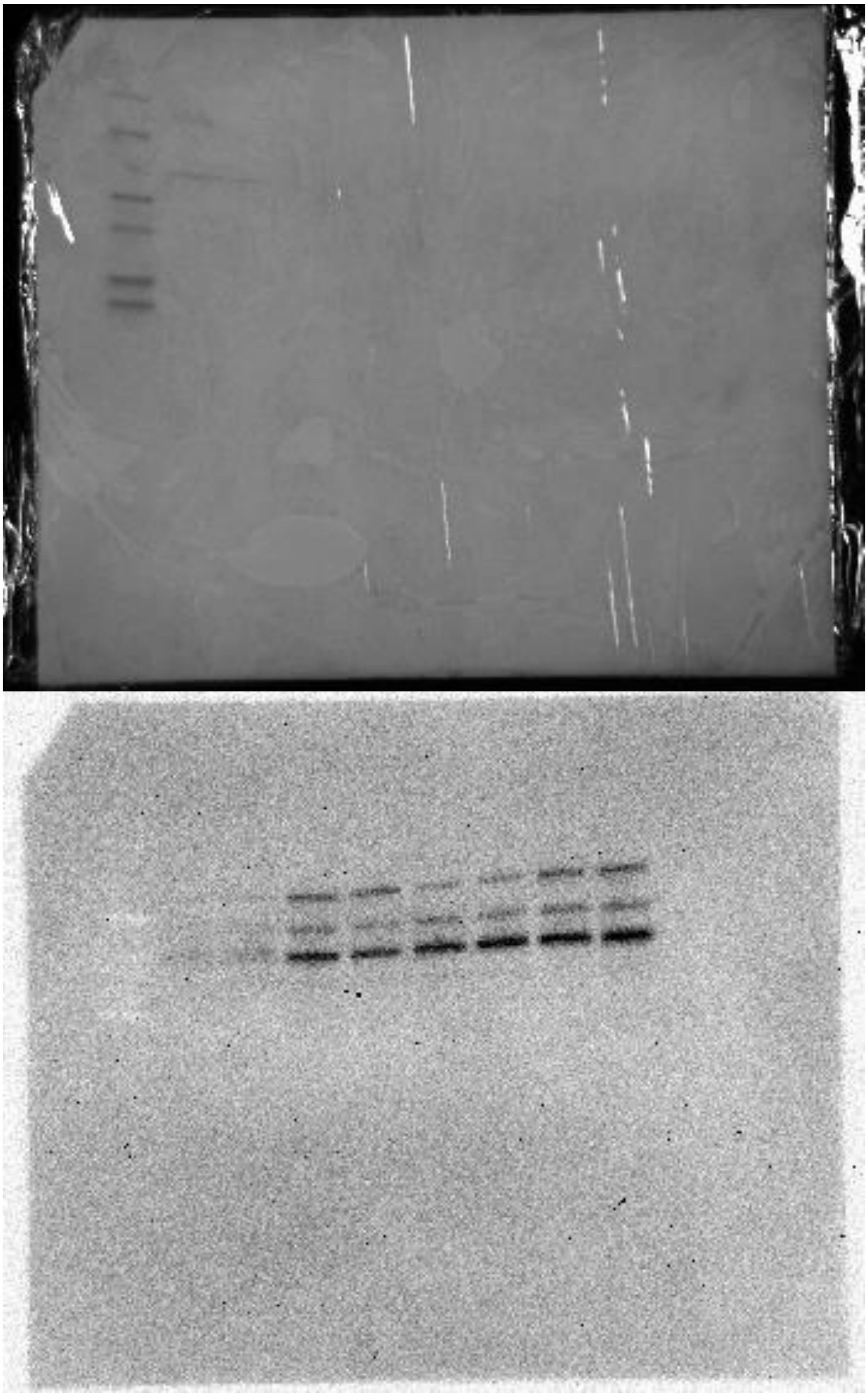

Fig S10. The uncropped Western blots of Fig 2e lower panel secreted proteins.

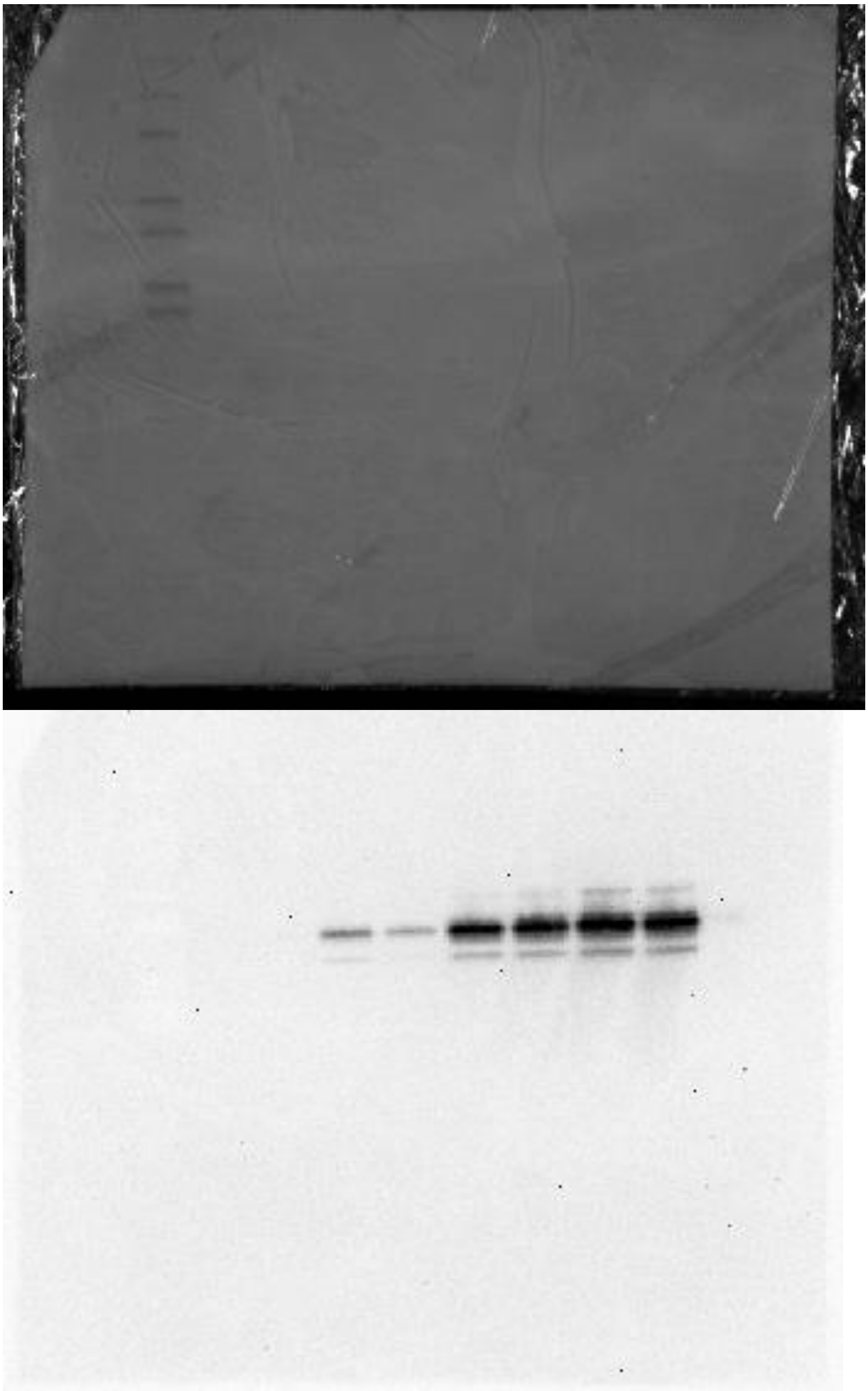

Fig S11. The uncropped Western blots of Fig 2f.

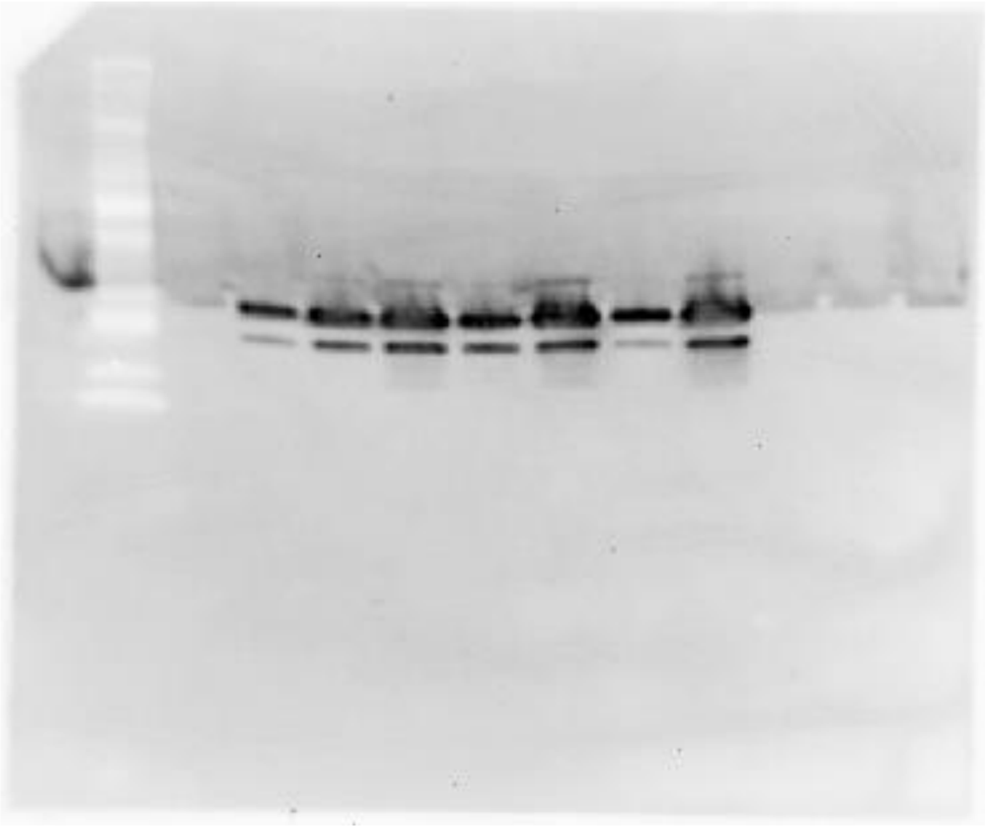

Fig S12. The uncropped silver staining gel of Fig S3.

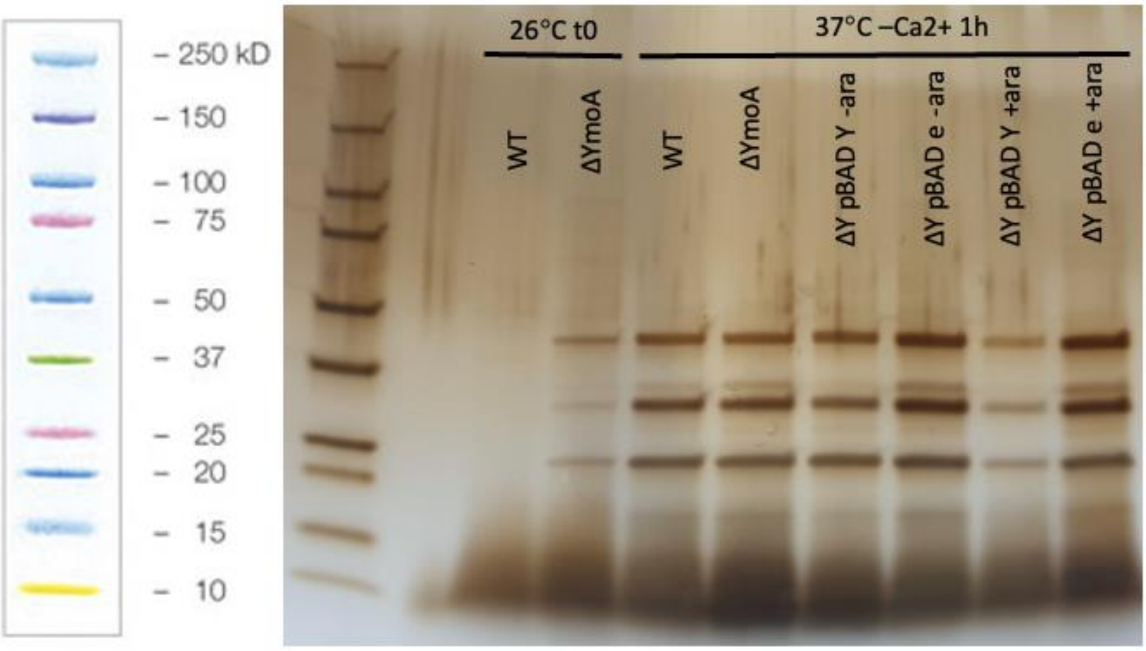

Fig S13. The uncropped silver staining gel of Fig S5.

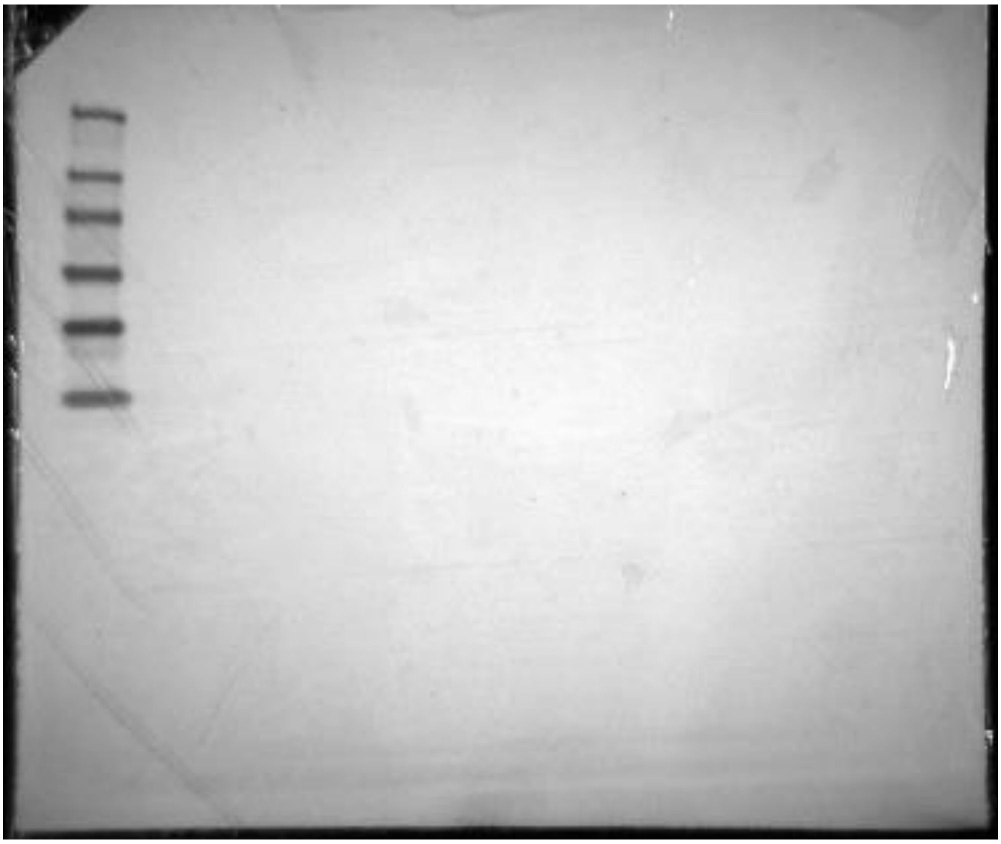

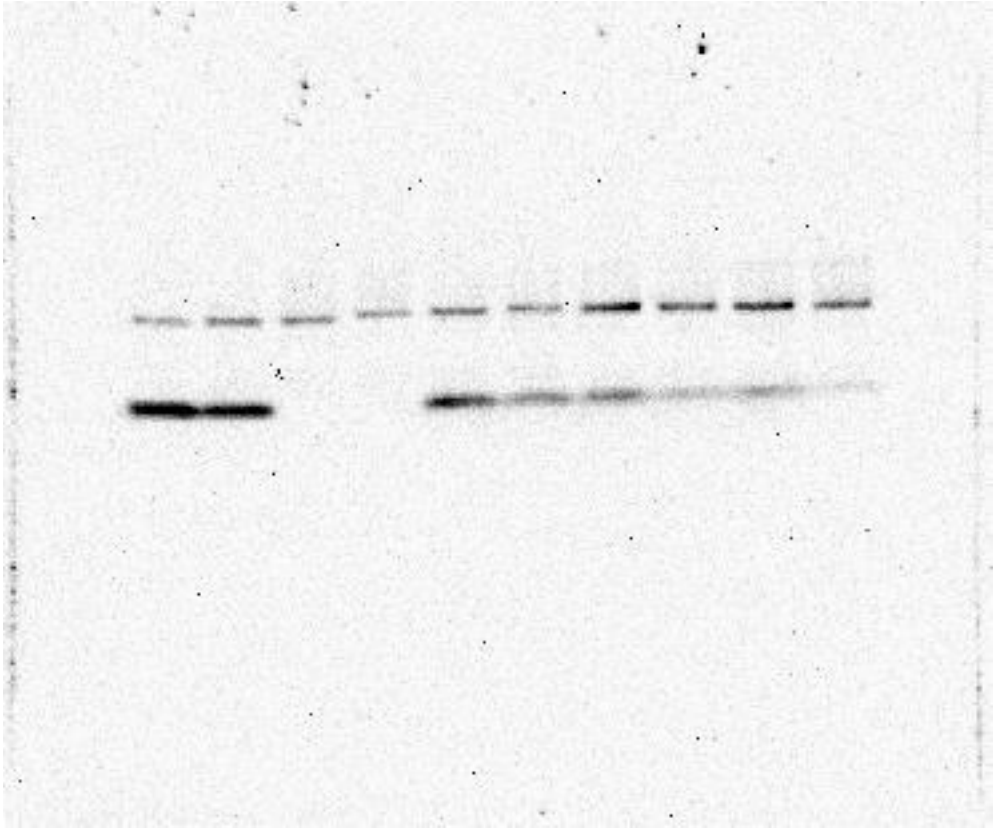

Table S1: 2nd way Anova statistical comparison of the bacterial growth rate presented in Figure 2c.

**Tukey's multiple comparisons test**	**Adjusted P Value**	**Summary**
26°C		
WT vs. *ΔymoB*	0,9620	ns
WT vs. Δ*ymoA*	<0,0001	****
WT vs. Δ*ymoB-ymoA*	<0,0001	****
ΔymoB vs. Δ*ymoA*	<0,0001	****
ΔymoB vs. Δ*ymoB-ymoA*	<0,0001	****
Δ*ymoA* vs. Δ*ymoB-ymoA*	0,8642	ns
37°C + Ca^{2+}		
WT vs. *ΔymoB*	0,3849	ns
WT vs. Δ*ymoA*	<0,0001	****
WT vs. Δ*ymoB-ymoA*	<0,0001	****
ΔymoB vs. Δ*ymoA*	<0,0001	****
ΔymoB vs. Δ*ymoB-ymoA*	<0,0001	****
Δ*ymoA* vs. Δ*ymoB-ymoA*	0,9938	ns
37°C - Ca^{2+}		
WT vs. *ΔymoB*	0,9705	ns
WT vs. Δ*ymoA*	0,0094	**
WT vs. Δ*ymoB-ymoA*	0,0015	**
ΔymoB vs. Δ*ymoA*	0,0032	**
ΔymoB vs. Δ*ymoB-ymoA*	0,0005	***
Δ*ymoA* vs. Δ*ymoB-ymoA*	0,8761	ns

Table S2: Ordinary one-way Anova statistical comparison of the bacterial growth rate presented in Figure 2d.

**Dunnett's multiple comparisons test**	**Adjusted P Value**	**Summary**
WT vs. p*ymoB*	0,4141	ns
WT vs. WT + p*ymoA*	0,0325	*
WT vs. p*ymoB-ymoA*	0,8301	ns
WT vs. ymoB	>0,9999	ns
WT vs. p*ymoB*	0,1888	ns
WT vs. p*ymoA*	0,0964	ns
WT vs. p*ymoB-ymoA*	>0,9999	ns
WT vs. Δ*ymoA*	<0,0001	****
WT vs. p*ymoB*	<0,0001	****
WT vs. Δ*ymoA* + p*ymoA*	0,0059	**
WT vs. p*tomB-ymoA*	>0,9999	ns
WT vs. -	<0,0001	****
WT vs. p*ymoB*	<0,0001	****
WT vs. p*ymoA*	<0,0001	****
WT vs. p*tomB-ymoA*	0,2920	ns

Table S3: Ordinary one-way Anova statistical comparison of the bacterial growth rate presented in Figure 5c.

**Dunnett's multiple comparisons test**	**Adjusted P Value**	**Summary**
ΔymoA + ymoA vs. WT	0,0045	**
ΔymoA + ymoA vs. Δ*ymoA*	<0,0001	****
ΔymoA + ymoA vs. WT + ymoA	0,0017	**
ΔymoA + ymoA vs. Δ*ymoA*+ *ymoA* R21A	<0,0001	****
ΔymoA + ymoA vs. Δ*ymoA* + *ymoA* H65A	<0,0001	****
ΔymoA + ymoA vs. Δ*ymoA* + *ymoA* K67A	<0,0001	****

Table S4: Ordinary one-way Anova statistical comparison of the bacterial growth rate presented in Figure 5d-f.

**Tukey's multiple comparisons test**	**Adjusted P Value 26°C**	**Adjusted P Value 37°C + Ca^{2+}**	**Adjusted P Value 26°C 300mM NaCl**
WT vs. Δ*ymoA*	<0,0001	<0,0001	<0,0001
WT vs. WT + *ymoA*	0,9481	0,9999	0,9999
WT vs. Δ*ymoA* + *ymoA*	0,0037	0,0060	0,0004
WT vs. Δ*ymoA*+ *ymoA* R21A	<0,0001	<0,0001	<0,0001
WT vs. Δ*ymoA* + *ymoA* H65A	<0,0001	<0,0001	<0,0001
WT vs. Δ*ymoA* + *ymoA* K67A	<0,0001	<0,0001	<0,0001
Δ*ymoA* vs. WT + *ymoA*	<0,0001	<0,0001	<0,0001
Δ*ymoA* vs. Δ*ymoA* + *ymoA*	<0,0001	<0,0001	<0,0001
Δ*ymoA* vs. Δ*ymoA*+ *ymoA* R21A	0,0119	0,0072	<0,0001
Δ*ymoA* vs. Δ*ymoA* + *ymoA* H65A	0,0033	<0,0001	<0,0001
Δ*ymoA* vs. Δ*ymoA* + *ymoA* K67A	0,9887	0,0010	<0,0001
WT + *ymoA* vs. Δ*ymoA* + *ymoA*	0,0007	0,0033	0,0007
WT + *ymoA* vs. Δ*ymoA*+ *ymoA* R21A	<0,0001	<0,0001	<0,0001
WT + *ymoA* vs. Δ*ymoA* + *ymoA* H65A	<0,0001	<0,0001	<0,0001
WT + *ymoA* vs. Δ*ymoA* + *ymoA* K67A	<0,0001	<0,0001	<0,0001
Δ*ymoA* + *ymoA* vs. Δ*ymoA*+ *ymoA* R21A	0,0031	<0,0001	<0,0001
Δ*ymoA* + *ymoA* vs. Δ*ymoA* + *ymoA* H65A	0,0111	0,0019	0,0027
Δ*ymoA* + *ymoA* vs. Δ*ymoA* + *ymoA* K67A	<0,0001	<0,0001	<0,0001
Δ*ymoA*+ *ymoA* R21A vs. Δ*ymoA* + *ymoA* H65A	0,9894	0,0966	0,2433
Δ*ymoA*+ *ymoA* R21A vs. Δ*ymoA* + *ymoA* K67A	0,0441	0,9173	0,7396
Δ*ymoA* + *ymoA* H65A vs. Δ*ymoA* + *ymoA* K67A	0,0121	0,4906	0,0185

Table S5. Primers used for the generation of deletion mutants.

Name	Sequence
YmoA fw	gcatgctagtaatgacaggccttctcctgcgg
YmoA rv	gccatacagtaggtggaattaaacgcatcaggtagtcagtttttgtcat
YmoB fw	gcatgcgtatttttaccccgatgactggatgattaatgaac
YmoB rv	gtacatacgtattccccgtcatgccgcttaggcgagtactcatccat

Table S6. Primers used for protein purification.

Name	Sequence
YmoA fw	atggatgagtactcgcctaagcggcat
YmoA_his_rv	ctaatgatgatgatgatgatgtttcacatgttg
YmoA_R21A fw	gatacgctagaagctgtaattgaaaaa
YmoA_R21A_rv	tttttcaattacagcttctagcgtatc
YmoA_H65A_fw	actgtatggcaagctgtgaaacat
YmoA_H65A_rv	atgtttcacagcttgccatacagt

Supplementary data. RNA-seq analysis is presented in Excel format.
