# Supplementary material for: YmoA functions as a molecular stress sensor in Yersinia
Source: Commun Biol. 2025 Feb 13;8:225. doi: 10.1038/s42003-025-07675-y (PMC11825884; doi:10.1038/s42003-025-07675-y)
Supplement: Supplementary file 3 — Description of Supplementary Data [file 42003_2025_7675_MOESM3_ESM.pdf]

Description of additional supplementary data

file name:Supplementary data.
Description: RNA-seq analysis is presented in Excel format.
